# D-dimer is an essential accompaniment of circulating tumor cells in gastric cancer

**DOI:** 10.1186/s12885-016-3043-1

**Published:** 2017-01-13

**Authors:** Dongmei Diao, Yao Cheng, Yongchun Song, Hao Zhang, Zhangjian Zhou, Chengxue Dang

**Affiliations:** 1 Oncology Surgery Department, First Affiliated Hospital of Xi’an Jiaotong University, 277 West Yanta Road, Xi’an, Shaanxi 710061 People’s Republic of China; 2 Thoracic Surgery Department, Second Affiliated Hospital of Xi’an Jiaotong University, Xi’an, People’s Republic of China

**Keywords:** D-dimer, Gastric cancer (GC), Metastasis, Circulating tumor cells (CTCs), Outcome

## Abstract

**Background:**

Fibrinogen (FIB) is an important source of fibrin, which plays a crucial role in circulating tumor cells (CTCs) extravasation and distant metastasis development. We hypothesize it’s stable final product, plasma D-dimer, may be associated with CTCs appearance and can reflect the metastatic phenotype in cancer patients.

**Methods:**

We first verified our hypothesis in different murine gastric cancer metastasis models in vivo, plasma D-dimer and fibrinogen as well as its degradation products were directly examined in three metastasis immune-deficient mouse models and in controls. Next, we gathered and analyzed the result of plasma D-dimer levels and CTCs numbers in 41 advanced primary gastric cancer (GC) patients. A follow-up study was conducted in these patients.

**Results:**

In three in vivo murine metastasis models, plasma D-dimer levels were extremely elevated in a hematogenous and intraperitoneal murine model of metastasis compared with a subcutaneous tumor model and the control group, supporting our previous hypothesis. While in 41 GC patients, the result displayed that plasma D-dimer levels were remarkably increased in patients with distant metastases, especially in visceral metastases patients. Additionally, linear association was shown between D-dimer level and CTCs numbers (*R* ^2^ = 0.688, *p* < 0.001), additionally, plasma D-dimer represent a better survival predictor than CTCs.

**Conclusions:**

Plasma D-dimer is an essential accompaniment of CTCs in GC that is easy to measure and lower in cost, and can be used in the detection of hematogenous metastasis.

## Background

Metastasis is beginning when primary tumor release tumor cells into circulatory system becoming CTCs [1]. After CTCs arrest and generated in vascular, they must working together with coagulant factors like platelets (PLT), FIB and other clotted plasma factors to form micro-thrombus to help them to adhere and transfer in distant organs [2]. Based on previous studies, they believe there was a close relationship between higher tumor-associated procoagulant activity state and tumor metastasis [2, 3]. Coagulation and fibrinolysis system activation in cancer patients, may partly reflects the diffusion of tumor cells in host circulatory system.

Fibrinogen (FIB) is an important source of fibrin, which plays a crucial role in circulating tumor cells (CTCs) extravasation and distant metastasis development [4, 5]. D-dimer, the final stable product of fibrin, which elevated after enhanced activation of Coagulation and fibrinolysis system, widely used in detect and exclude deep vein thrombosis and associated thromboembolic diseases [6–8]. Recent years, several studies reported that plasma D-dimer elevated in malignant tumors and its expression levels positively correlates with an advanced tumor stage, overall survival and therapy response [9–16]. In our previous study in GC patients, we found, D-dimer can better predict asymptomatic visceral metastasis than FIB and other factors [16]. Although FIB is essential in CTCs survival, but not strong in clinical use to detect metastasis, but D-dimer revealed its advantages in this field. It may be associated with CTCs appearance and can reflect the metastatic phenotype of caner patients.

The overall survival in advanced tumor stage in GC patients is not so optimistic in present study of the word, so early diagnose of metastasis is an important step in GC patients. We designed this multiple experimental study based on previous research to found If it is a reflector when CTC arise in circulation and the direct association of circulating tumor cells and D-dimer, we also want to explore the effectiveness of the management of metastasis by plasma D-dimer and compared with other factors like CellSearch system detected CTCs in GC patients. In this study, we first verified our hypothesis in different murine models in vivo. We also tested the in vivo levels of fibrinogen (FIB) and fibrin degradation products (FDP). Then, we collected data from blood samples of advanced GC patients to evaluate the clinical use of D-dimer in the detection of hematogenous metastasis and to determine the correlation with CTCs. CTC counts in this study were detected by the CellSearch system with specific antibodies, CTC cells was detected with CK (cytokeratins8, 18, and/ or 19) (+) and DAPI(+) and CD45(−).

## Methods

### Cell culture

The human GC cell lines (MGC80-3 (TCHu 84), AGS (TCHu232)) obtained from Type Culture Collection of Shanghai Chinese Academy of Sciences which conserved in our lab, the cells were cultured in mediem (DMEM, Hyclone, Logan, UT, USA) supplemented with fetal calf serum (10% concentration, Hyclone, Logan, UT, USA).

### Animal experiments

To better understand the relationship between plasma D-dimer levels and the progression of human GC, we injected human GC-derived cells into immune-deficient mice; The male immune-deficient mice purchased from Beijing Laboratory Animal Center, 5 × 10^5^ GC cells (AGS and MGC80-3) were separated and injected into the mice subcutaneously to induce subcutaneous metastasis, into the abdominal cavity to induce intra-peritoneal metastasis, and into mice tail vein to induce hematogenous metastasis, respectively. The control mice were injected with 200 μl of PBS. After 2 weeks, murine blood was harvest from the eyeball and placed in the tube with heparin lithium-anticoagulant. At the same time, we also performed dissections of these mice and the lungs were removed to detect hematogenous metastasis.

### Patients

GC patients diagnosed by a pathologist according to an endoscopic biopsy and who were hospitalized at the First-Affiliated-Hospital of medicine collage, Xi’an Jiaotong University between 1st Jan 2009 and 1st Jan 2013 were enrolled in this study. Anyone who had history of venous thrombosis or received any anti-coagulation treatment was excluded. Also excluding principle involved cerebrovascular or cardiovascular disease, inflammatory diseases, history of malignancies, and those who received previous anticancer treatments were also excluded. After exclusion of the above-mentioned patients, a total of 41 GC patients were involved in our study, Of which all the patients received similar chemotherapy treatment. (5-fluorouracil,5-Fu, oxaliplatin, OXA and folinic acid, 4–8 times; at the interval of 2 weeks) after nearly 3 weeks recovery of surgery, except one patients only receive 2 times before dead and the overall survival is 2 month. 29 patients received treatment in First Affiliated Hospital of medicine college of Xi’an Jiaotong University accepted continues follow-up by telephone or in hospital. All the follow-up terminated in Jan 2015 as defined >2 year. There were two additional end-point referring to tumor recurrence and pass away. Cancer stage was defined in the accordance of American Joint Committee on Cancer-7 (AJCC-7).

### D-dimer assays

Venous blood was collected in sodium citrate tubes from each sample for D-dimer detecting. The amount of FDP, FIB, D-dimer and CEA were assayed using ELISA detection. In clinical detection, latex-enhanced immunoturbidimetric assay was also used to analysis the level of FIB. FDP and D-dimer, which were the same as our previous experiment [15], all the samples were collected when they first get pathological diagnosis before any treatments. D-dimer level in normal human plasma is usually below 1.0 μg/ml.

### CTC counts

Following the instruction of CellSearch system (Veridex LLC, Warren, NJ, USA), blood CTCs were isolated by immunomagnetic, and then, immune-fluoresence-staining was employed to detected CTCs positive cells in the mechanism that CTCs were identified by lacking CD45 which express cytokeratin (8, 18, and/ or 19). The method and criteria of defining tumor cell was the same with instructed previously [17]. 7.5 ml of blood of each patient enrolled was accumulated to detect CTCs, and the detail of CellSpotter & CellSearch was all discussed by Allard, et all [17]. As previous reported, 2 CTCs or more detected in 7.5 ml blood is defined as CTCs positive [17]. Based on this criterion, more than 2 CTCs in one blood sample was defined as positive in this study.

### Statistical analysis

All the data was analyzed using analysis software SPSS 13.0 (IL, USA). The data from in vivo was present in the form of SE, that is mean ± standard error, two-tailed Students’ *T*-test and multiple comparison in ANOV were used. For distribution of plasma D-dimer in GC patient was not in normal way, so quartile range (Q) and median (M) were used to report it. Also, Spearman correlation analysis was used to evaluate univariate analysis. In order to identify any independent variables about D-dimer, multiple linear regression models also was selected. Survival were analyzed by log-rank tests, and survival curves was formed in the help of Kaplan-Meier method The statistically significant was defined as *p* < 0.05.

## Result

### Plasma D-dimer in vivo model

**Fig. 1:**
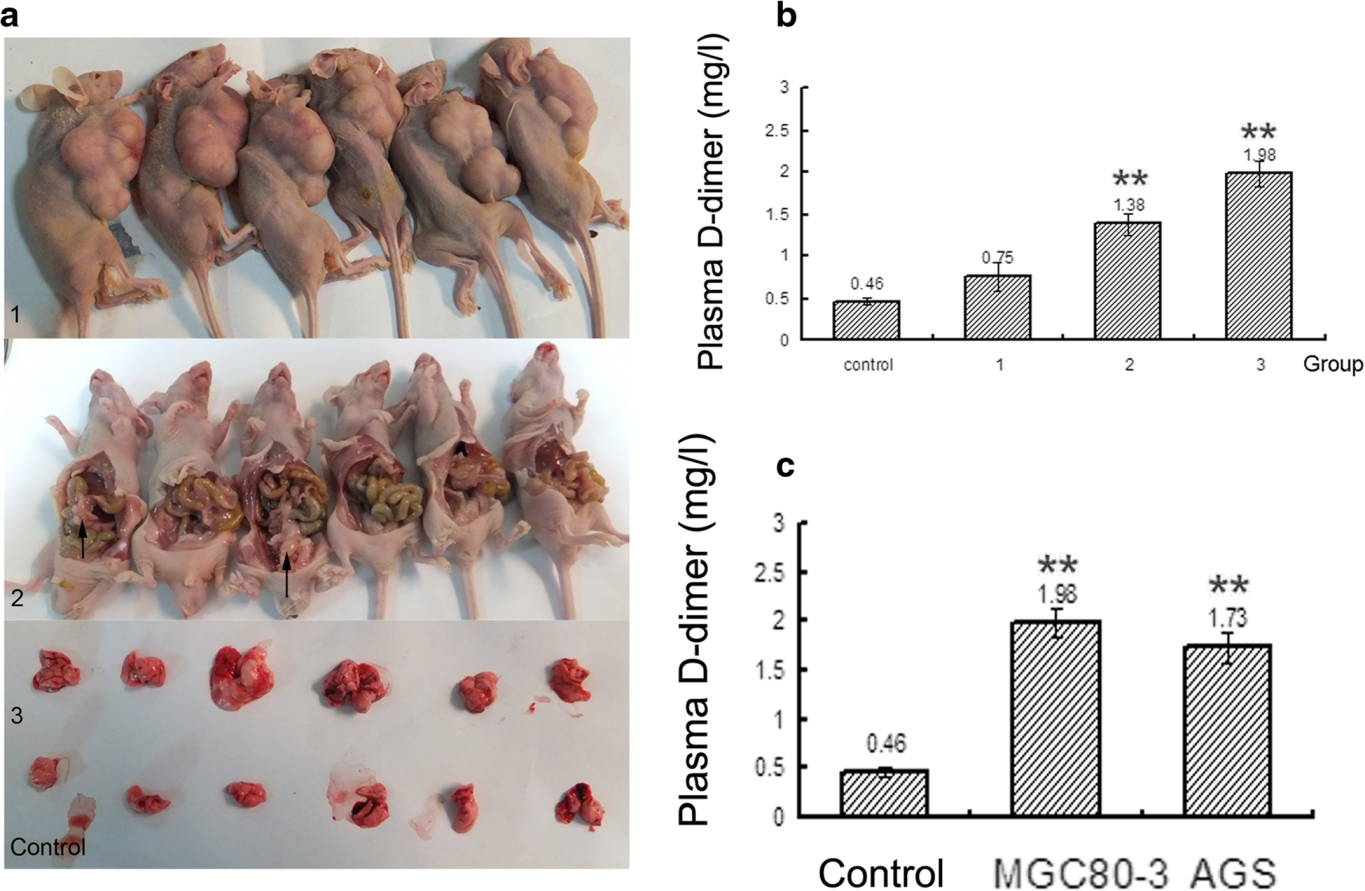
Plasma D-dimer levels in different in vivo metastasis models. **a**, Different in vivo metastasis models: 1. subcutaneous metastasis, 2. intra-peritoneal metastasis, 3. hematogenous metastasis and control. **b**, The plasma D-dimer levels increased gradually in the different in vivo models compared with the control, hematogenous (3) > intra-peritoneal (2) > subcutaneous (1) > control (*p* < 0.001). **c**, The plasma D-dimer levels in the hematogenous metastasis in vivo model were higher inMGC80-3 GC cell lines and AGS cell lines compared with the control group (*p* < 0.001). * indicates that the correlation is significant at the 0.05 level (2-tailed). ** indicates that the correlation is significant at the 0.01 level (2-tailed)

To clarify and define the whether there has any relation between development of GC progression and plasma D-dimer, we injected different human GC cell lines (AGS and MGC80-3) into immunodeficient mice. After two weeks, it was observed that the D-dimer levels were gradually elevated in subcutaneous metastasis (0.75 mg/l ±0.17), intra-peritoneal metastasis (1.38 mg/l ± 0.13) and hematogenous metastasis (1.98 mg/l ± 0.15), which is higher than control group mice (0.46 mg/l ± 0.05) (*p* < 0.001) (Fig. 1a, b). Moreover, the results strongly supported that the plasma D-dimer were exceptionally increased in the hematogenous metastasis group (MGC80-3, 1.98 mg/l ± 0.15) than in PBS treatment group (0.46 mg/l ± 0.05,*p* < 0.001) and D-dimer level was also elevated in hematogenous metastasis group (AGS, 1.73 mg/l ± 0.15) compared with the PBS control group too (0.46 mg/l ± 0.05) (*P* < 0.001) (Fig. 1c).

### Levels of FIB and FDP in vivo model

**Fig. 2:**
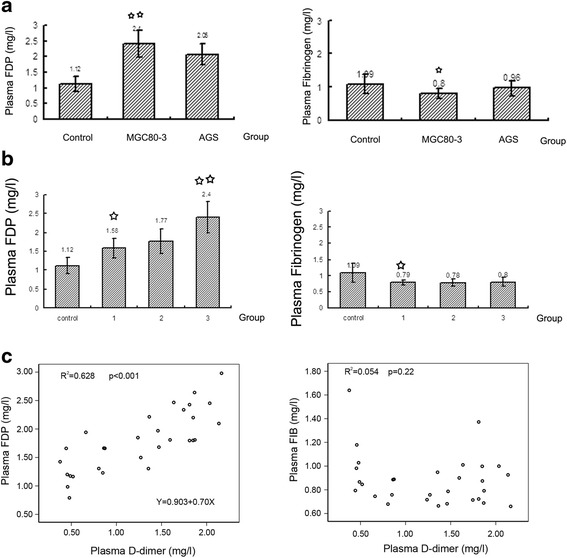
Plasma FIB and FDP levels in different in vivo metastasis models. **a**, **b**, The plasma FDP levels were increased in different in vivo models, but not to the same extent as the D-dimer levels; The plasma FIB levels in the different in vivo models were decreased in the subcutaneous model compared with the control, but were not different among the several tumor models. **c**, In different in vivo models, the plasma D-dimer level showed a linear relationship with plasma FDP, but not with plasma FIB

Although the development of plasma FDP was consistent with that of plasma D-dimer, it was not as obvious as the presence of D-dimer (Fig. 2a, b). However, plasma FIB was decreased in the three tumor-burdened groups, but no differences were found between the groups (Fig. 2a, b). Plasma FDP showed a linear association with the D-dimer level (*R* ^2^ = 0.628, *p* < 0.001), and this trend can be seen in Fig. 2a. However, FIB did not show any considerable association with the plasma D-dimer level (Fig. 2c).

### Patient data

In total, 41 patients with gastric cancer (34 male and 7 female with the age at 40–83 years) were enrolled in this study. The gastric cancer group enrolled 11 stage III B and 30 stage IV patients. Among all the patients,14 patients were defined via histological grade as G1 stage, that is well differentiated, G2 stage is moderately differentiated, moreover, another 27 patients were classified with G3, that is poorly differentiated or G4 as undifferentiated. After clinical multi-departmet-analysis and evaluation, the following 41 patients with GC with subgroup as: 20 patients received surgical resection without margins regent, that is R0 resection, 15 patients received gastric resection and more than 20 lymph node with microscopic residual in pathology result (R1 resection) and 6 patients received palliative resection accompany or not with exploratory laparotomy. Data before treatments of D-dimer, CEA and CTCs are listed in Table 1.

**Table 1:** The basic patient data and median and 25th–75th percentile of plasma D-dimer, CEA levels and CTC positive rate in 41 Gastric Cancer patients

Variable	No of case	CTC-positive (%)	D-dimer	CEA
Gender				
Male	34	10 (29.41%)	1.10 (0.58–2.35)	2.80 (1.58–19.53)
Female	7	3 (42.85%)	1.30 (0.50–3.30)	4.21 (1.63–19.46)
Age				
≤ 61	19	7 (36.84%)	1.30 (0.50–3.50)	2.79 (1.38–19.46)
>61	22	6 (27.27%)	1.05 (0.70–1.77)	3.15 (1.70–10.25)
Histological Grade				
G1–G2	14	4 (28.57%)	0.85 (0.48–1.62)	4.27 (2.92–25.30)
G3–G4	27	9 (33.33%)	1.30 (0.70–3.30)	2.25 (1.38–5.46)
TNM Stage				
IIIB	11	0	0.50 (0.30–1.10)	4.34 (2.16–19.46)
IV	30	13	1.50 (0.87–3.35)	2.43 (1.58–10.25)
Metastasis				
M0	20	3	0.95 (0.50–1.65)	2.60 (1.39–14.59)
M1	16	6	1.10 (0.62–2.12)	2.99 (1.78–39.44)
M2	5	4	7.10 (2.45–26.80)	4.70 (1.56–64.81)
Surgery				
R0	20	3	1.00 (0.35–1.65)	2.50 (1.39–4.76)
R1	15	7	1.60 (0.70–3.80)	2.96 (1.63–17.83)
Other	6	2	1.40 (0.80–9.35)	28.11 (2.40–157.25)

*M1* means intra-peritoneal metastasis; *M2* means visceral metastasis

Correlations of the D-dimer, CEA & CTCs with other important factors are recorded and listed (Table 2). Spearman correlation analysis demonstrate the plasma D-dimer and CTCs have greatly relationship with metastatic lymph-node invasion (*p* = 0.014 & *p* = 0.021), whereas the CEA level did not demonstrate any correlation with metastasis (*p* = 0.315) as shown in Table 2.

**Table 2:** Associations between D-dimer, CEA, CTC and Clinicopathological Features in Patients with Gastric Cancer

Variable	*P* (CTC)	P (D-dimer)	*P* (CEA)
Gender	0.424	0.973	0.721
Age	0.589	0.799	0.119
Histological Grade	0.513	0.235	0.07
TNM Stage	0.012*	0.002**	0.487
Metastasis	0.014*	0.021*	0.315

*P* indicate that the *p*-value was analyzed via Spearman Correlation analysis was employed; *indicates *p*-value < 0.05 (2-tailed). **indicates *p*-value < 0.01 (2-tailed)

#### The association between D-dimer and CTCs

**Fig. 3:**
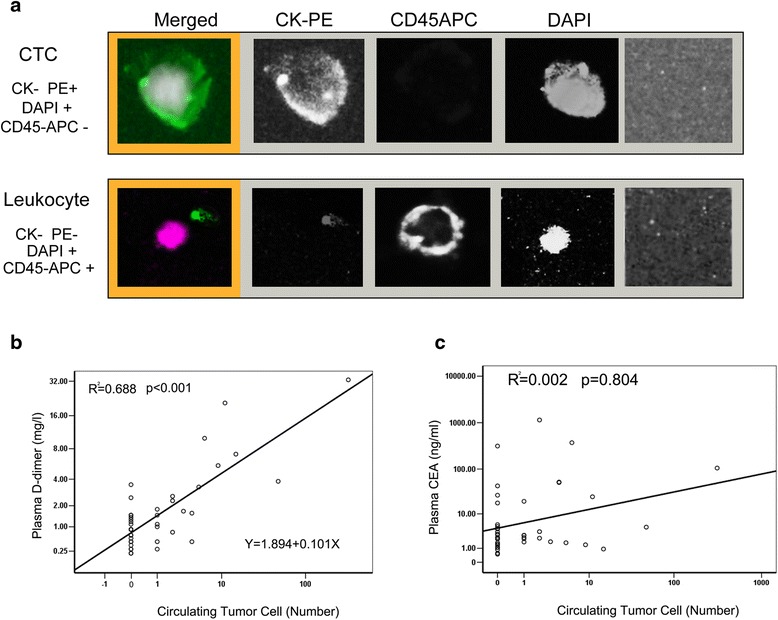
The images of CTC and leukocyte and relationship among the CTCs and the levels of plasma D-dimer and CEA. **a**, The images of CTC and leukocyteB detected by Cellsearch system in this study. **b**, The number of CTCs showed a linear relationship with the plasma D-dimer levels. **c**, The CTCs counts did not showed a linear relationship with the plasma CEA levels

CTC counts in this study were performed with the CellSearch system with specific antibodies: CTC cells was detected wtih CK (cytokeratins8, 18, and 19) (+) and DAPI(+) and CD45(−); Leukocyte cells were detects with CK(−) and DAPI(+) and CD45(−) (Fig. 3a). In this 41 patients with advanced GC (including 11 stage III B and 30 stage IV patients) 19 of the 41 patients had detectable CTCs ≥ 1 (46.3%), 13 of 41 patients had detectable CTCs ≥ 2 (31.7%), and 10 of the 41 patients had detectable CTCs ≥ 3 (24.4%). The result strongly exhibited a liner relationship between CTCs and D-dimer. (*R* ^2^ = 0.688, *p* < 0.001), but not with CEA levels (*R* ^2^ = 0.002, *p* < 0.804) (Fig. 3b, c).

**Fig. 4:**
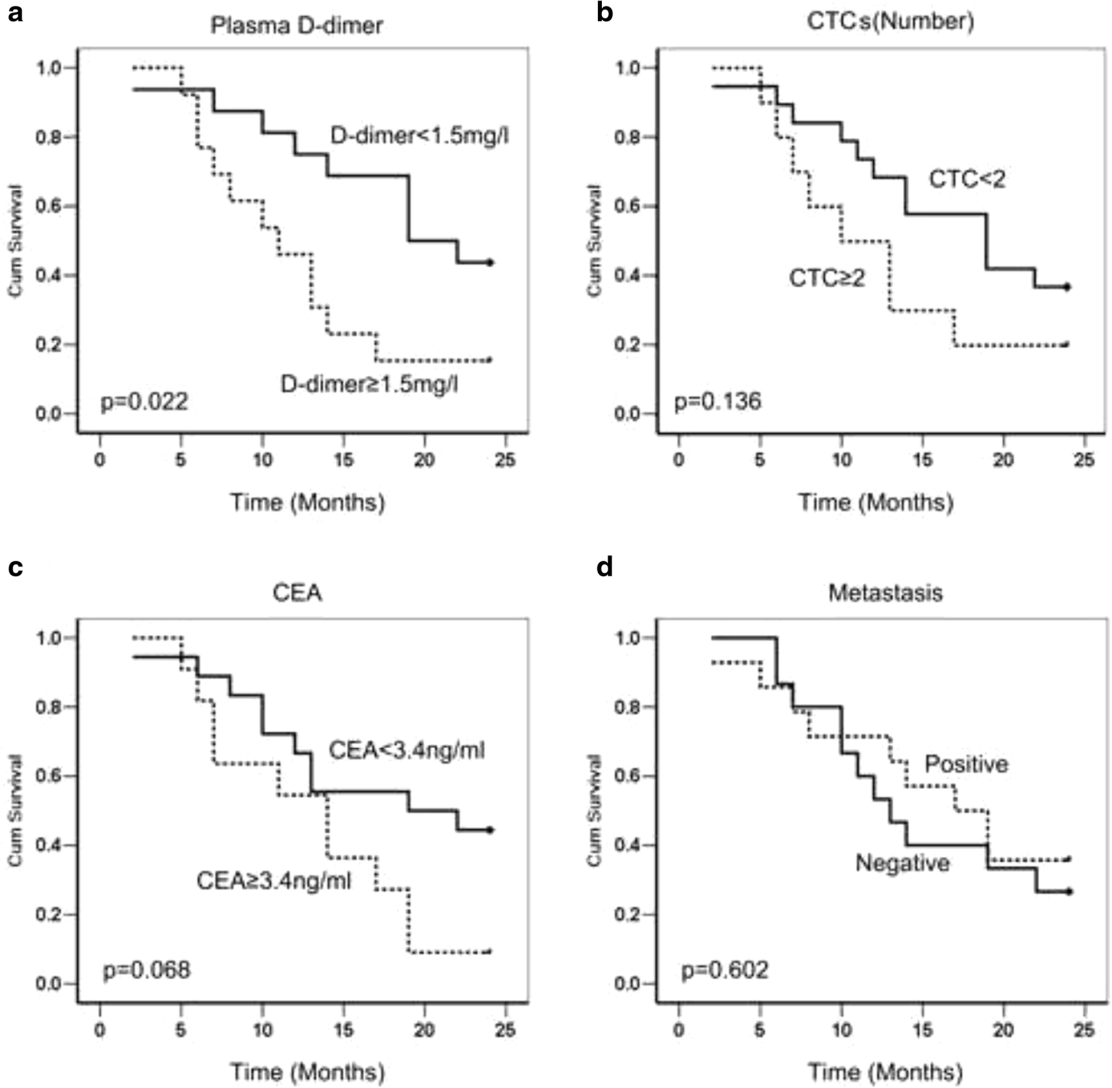
The Kaplan-Meier analysis of the survival of patients with gastric cancer. **a**, The survival rate was lower in patients with D-dimer levels over 1.5 mg/l (A), but the levels of CEA (**b**) and CTC (**c**) and Metastasis (**d**) diagnosed by imaging did not show any significant difference in the survival analysis

We compared the effectiveness of the plasma D-dimer, CTC, CEA and metastasis (detected by imaging tests and/or by the pathology) in the prediction of patient outcomes. Different levels (high/low) were defined in the standard of baseline plasma D-dimer amount (≥1.5 or < 1.5 mg/ml), CTCs (≥2 or <2), and the CEA level (≥3.4 ng/ml or <3.4 ng/ml) and Metastasis (as detected by imaging tests and/or by the pathology) (positive or negative) in 29 followed-up advanced GC patients, the result found that the D-dimer level (*p* = 0.022) had a higher accuracy in prognosis prediction than CTCs (*p* = 0.136), CEA (*p* = 0.068) and even metastasis (*p* = 0.602) in advanced GC patients (Table 3 and Fig. 4a–d).

**Table 3:** Prognostic variables of D-dimer (mg/l), CEA, CTC for overall survival in GC patients

Prognostic Variable	No. case (survival rate)	Median survival time (m)	95% CI	*P*
D-dimer				
≥ 1.5 mg/l	13 (15.4%)	11	6.6–21	0.022*
<1.5 mg/l	16 (43.8%)	19	11.2–26.8	
CTC				
≥ 2	10 (20.0%)	10	4.8–15.2	0.136
<2	19 (36.8%)	19	11.9–26.0	
CEA				
≥ 3.4 μg/ml	11 (9.1%)	14	6.7–21.3	0.068
<3.4 μg/ml	18 (44.4%)	19	0.29–37.7	
Metastais				
Negative	15 (26.7%)	13	9.2–16.8	0.602
Positive	14 (35.7%)	17	10.9–23.1	

*P* indicate that the *p*-value was analyzed via Kaplan-Meier method; *indicates *p*-value <0.05 (2-tailed). *CI* means confidence interval of median survival time

## Discussion

**Fig. 5:**
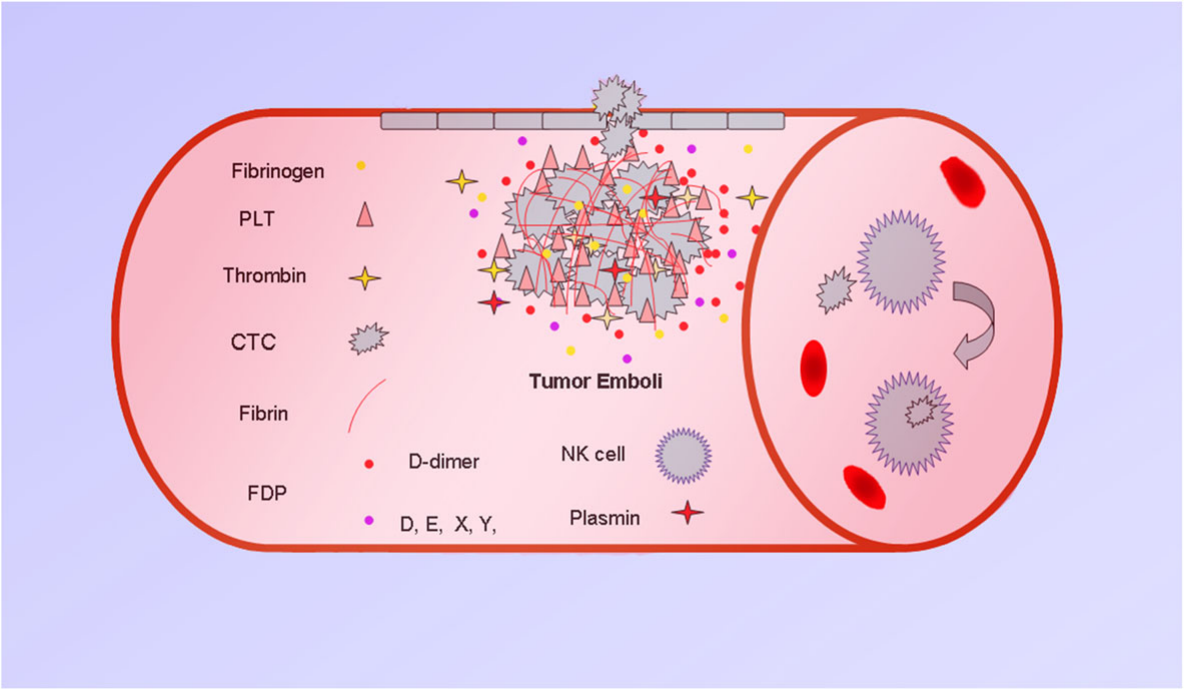
The potential mechanism of D-dimer elevation in malignancies. Clots (tumor emboli) participate in the process of metastasis through a variety of mechanisms including the following: the protection of cancer cells from the destruction of the immune system, or from the physical stress of blood flow, the facilitation of the attachment of tumor cells to the vessel walls, the enhancement of extravasation or angiogenesis. The components of the coagulation system including platelets, fibrin, thrombin, plasmin, tissue factor, sFn, and PARs, among others, have been shown to promote clot formation, which can affect metastasis. Fibrinogen plays a role in the adhesion and survival of circulating tumor cells. Fibrinogen is actively converted into fibrin and that process of fibrinolysis results in increased D-dimer levels. The plasma D-dimer levels are elevated after clot formation and may be involved in the promotion of a metastatic phenotype in the bloodstream

Nowadays, distant metastasis remains poor prognosis and leads to invalid treatment in cancer patients. It is widely accepted that activated coagulation, which is common in malignancy, provides great facile for cancer metastasis [2, 18–21]. Moreover, in cancer patients, it usually observed evidence of hemostatic system activation, which is a well-known phenomenon, and studies in recent years have shown that cancer and hemostatic system exist a bidirectional effect. Potential mechanism of D-dimer elevation in malignancy might associated with CTCs Clot (tumor emboli): Following initial tumor cell arrest in the capillary or bigger vessel of an organ, then platelets, plasma and other elements are easily clotted and formed stably adhesion to the cancer cell through the generation of a thrombus that protects the cancer cells to proliferation, adhesive and spread via the vasculature. Clots (tumor emboli) participate in the process of metastasis in several mechanisms including the following: the protection for cancer cells from the destruction of the immune system, blood flow stress, the facilitation of the attachment of tumor cells to the vessel walls, the enhancement of extravasation or angiogenesis or the facilitation of endothelial cell retraction [2, 18–21]. The individual components of coagulation have been shown to affect metastasis. Palumbo et al. showed that FIB served as a significant factor for CTCs metastasis ability [22, 23]. The absence of platelets induced by anti-platelet agents or special gene modified mice that can not generate platelets decreased cancer cell metastasis potential [20, 24]. Metastasis was also reduced by anti- thrombin treatments, in addition, mice absence of platelet thrombin receptor also harbor less cancer metastasis [25]. FIB in the other hand, format fibrin and decomposed into fibrinolysis in some conditions, lead to elevated plasma D-dimer. In the case of CTCs clot forming, D-dimer usually increased, and this factor may reflect metastasis of cancer in bloodstream, as shown at Fig. 5.

In our present study, we first verified that plasma D-dimer are significantly increased in a metastasis model in vivo especially in hematogenous metastasis, underlying D-dimer increased when CTCs spread into the vascular, this factor reflect cancer metastasis and might be an accompaniment for CTCs of GC. Well in addition, we compared effectiveness of the levels of D-dimer, CellSearch-CTCs, CEA and Metastasis (as detected by imaging tests and/or by the pathology) in the prediction of patient outcomes and found plasma D-dimer served as a better outcome predictor comparing to CellSearch-CTCs detection in cost performance, CEA and metastasis in patients with advanced GC. What is very interesting, Graph describing D-dimer and Cell Search-CTCs showed a linear relationship. This work found D-dimer is an important accompaniment of CTCs which could be considered in the detection of hematogenous metastasis in gastric cancer in clinical.

Discovery and identification of effective cancer biomarkers predicting metastasis will be helpfully benefit for improving strategies of cancer treatment. Histopathological analysis of early stage hematogenous metastases in human frequently reveals the coexistence of thrombosis, with abundant fibrin deposition, coagulation factors is easily occupied for metastasis detection in clinical work. In our in vivo experiments, data showed that there was no markedly differences plasma FIB levels among different cancer groups. Even FIB is an essential factor in CTCs survival and metastasis, however, it is not yet feasible to test for metastasis in a clinical setting. While, detection plasma level of D-dimer supply with this advantage in the bottleneck of insufficient cost performance. FDP includes other degeneration products of fibrinogen and D-dimer, a linear association was also detected because affected by D-dimer. However, it was regret that CTCs counts in mouse model can not be detected by Cellsearch method because of the low volume of mouse blood, but in this clinical study in 41 GC patients, CTCs showed a linear association with plasma D-dimer (R2 = 0.688, *p* < 0.001), CTCs, we thought D-dimer can reflect CTCs number sometimes in human being, which may be also persuasive.

Cellsearch system can be used in CTCs detection in whole blood. A study by Khoury discovered an obvious correlation between CTC positive number and plasma D-dimer. increases D-dimer levels versus CTC counts are better suggesting progressive cancer state in 28 prostate cancer patients [26]. Present study did not find enough evidence directly support that Cellsearch-CTCs can be served as a good biomarker of human GC, because CTCs lack cytokeratin expression or as epithelial-mesenchymal transition (EMT) exist in cancer cell metastasis. Our study detected CTCs in 41 advanced GC patients by CellSearch System, and the result confirmed the linear relationship between CTCs and D-dimer in GC patients. We also found that the D-dimer level in the plasma was able to predict survival, which is more efficient than Cellsearch-CTCs detection, imaging methods and blood CEA. This finding convinced us that D-dimer can predict hematogenous metastasis, reflecting a negative outcome of patients with GC.

## Conclusions

In summary, we discussed that D-dimer, coming from decomposition of fibrin, stably exist in human plasma, served as an essential accompaniment of CTCs in GC that is easy to measure and lower in cost, and can be used in the detection of hematogenous metastasis. This detection method is of considerable value for routine test and can remarkably help clinical doctors predict GC patients outcome.

## Abbreviations

5-Fu: 5-fluorouracil
CEA: Carcino-embryonic antigen
CTCs: Circulating tumor cells
DIC: Disseminated intravascular coagulation
EMT: Epithelial-mesenchymal transition
FDP: Fibrin degradation products
FIB: Fibrinogen
GC: Gastric cancer
OXA: Oxaliplatin

## References

[1] Gupta GP, Massague J (2006). Cancer metastasis: building a framework. Cell.

[2] Im JH, Fu W, Wang H, Bhatia SK, Hammer DA, Kowalska MA (2004). Coagulation facilitates tumor cell spreading in the pulmonary vasculature during early metastatic colony formation. Cancer Res.

[3] Gay LJ, Felding-Habermann B (2011). Contribution of platelets to tumour metastasis. Nat Rev Cancer.

[4] Palumbo JS, Talmage KE, Massari JV, La Jeunesse CM, Flick MJ, Kombrinck KW (2005). Platelets and fibrin(ogen) increase metastatic potential by impeding natural killer cell-mediated elimination of tumor cells. Blood.

[5] Biggerstaff JP, Weidow B, Vidosh J, Dexheimer J, Patel S, Patel P (2006). Soluble fibrin inhibits monocyte adherence and cytotoxicity against tumor cells: implications for cancer metastasis. Thromb J.

[6] Righini M, Perrier A, De Moerloose P, Bounameaux H (2008). D-Dimer for venous thromboembolism diagnosis: 20 years later. J Thromb Haemost.

[7] Ay C, Vormittag R, Dunkler D, Simanek R, Chiriac AL, Drach J (2009). D-dimer and prothrombin fragment 1 + 2 predict venous thromboembolism in patients with cancer: results from the Vienna Cancer and Thrombosis Study. J Clin Oncol.

[8] Dompmartin A, Ballieux F, Thibon P, Lequerrec A, Hermans C, Clapuyt P (2009). Elevated D-dimer level in the differential diagnosis of venous malformations. Arch Dermatol.

[9] Go SI, Lee MJ, Lee WS, Choi HJ, Lee US, Kim RB (2015). D-dimer Can serve as a prognostic and predictive biomarker for metastatic gastric cancer treated by chemotherapy. Medicine (Baltimore).

[10] Tomimaru Y, Yano M, Takachi K, Kishi K, Miyashiro I, Ohue M (2006). Plasma D-dimer levels show correlation with number of lymph node metastases in patients with esophageal cancer. J Am Coll Surg.

[11] Lippi G, Franchini M, Biasiutti C, Dellagiacoma G, Salvagno GL, Guidi GC (2007). Increased D-dimer value and occult cancer in the absence of detectable thrombosis. Haematologica.

[12] Ay C, Dunkler D, Pirker R, Thaler J, Quehenberger P, Wagner O (2012). High D-dimer levels are associated with poor prognosis in cancer patients. Haematologica.

[13] Stender MT, Larsen TB, Sorensen HT, Thorlacius-Ussing O (2012). Preoperative plasma D-dimer predicts 1-year survival in colorectal cancer patients with absence of venous thromboembolism (VTE): a prospective clinical cohort study. J Thromb Haemost.

[14] Tas F, Ciftci R, Kilic L, Bilgin E, Keskin S, Sen F (2012). Clinical and prognostic significance of coagulation assays in melanoma. Melanoma Res.

[15] Diao D, Zhu K, Wang Z, Cheng Y, Li K, Pei L (2013). Prognostic value of the D-dimer test in oesophageal cancer during the perioperative period. J Surg Oncol.

[16] Diao D, Wang Z, Cheng Y, Zhang H, Guo Q, Song Y (2014). D-dimer: not just an indicator of venous thrombosis but a predictor of asymptomatic hematogenous metastasis in gastric cancer patients. PLoS One.

[17] Allard WJ, Matera J, Miller MC, Repollet M, Connelly MC, Rao C (2004). Tumor cells circulate in the peripheral blood of all major carcinomas but not in healthy subjects or patients with nonmalignant diseases. Clin Cancer Res.

[18] Horowitz NA, Blevins EA, Miller WM, Perry AR, Talmage KE, Mullins ES (2011). Thrombomodulin is a determinant of metastasis through a mechanism linked to the thrombin binding domain but not the lectin-like domain. Blood.

[19] Labelle M, Begum S, Hynes RO (2011). Direct signaling between platelets and cancer cells induces an epithelial-mesenchymal-like transition and promotes metastasis. Cancer Cell.

[20] van den Berg YW, Osanto S, Reitsma PH, Versteeg HH (2012). The relationship between tissue factor and cancer progression: insights from bench and bedside. Blood.

[21] Zhang W, Dang S, Hong T, Tang J, Fan J, Bu D (2012). A humanized single-chain antibody against beta 3 integrin inhibits pulmonary metastasis by preferentially fragmenting activated platelets in the tumor microenvironment. Blood.

[22] Palumbo JS, Kombrinck KW, Drew AF, Grimes TS, Kiser JH, Degen JL (2000). Fibrinogen is an important determinant of the metastatic potential of circulating tumor cells. Blood.

[23] Palumbo JS, Potter JM, Kaplan LS, Talmage K, Jackson DG, Degen JL (2002). Spontaneous hematogenous and lymphatic metastasis, but not primary tumor growth or angiogenesis, is diminished in fibrinogen-deficient mice. Cancer Res.

[24] Ware J (2012). Fragmenting the platelet to reduce metastasis. Blood.

[25] Camerer E, Qazi AA, Duong DN, Cornelissen I, Advincula R, Coughlin SR (2004). Platelets, protease-activated receptors, and fibrinogen in hematogenous metastasis. Blood.

[26] Khoury JD, Adcock DM, Chan F, Symanowski JT, Tiefenbacher S, Goodman O (2010). Increases in quantitative D-dimer levels correlate with progressive disease better than circulating tumor cell counts in patients with refractory prostate cancer. Am J Clin Pathol.

